# Effects of hydrothermal factors and human activities on the vegetation coverage of the Qinghai-Tibet Plateau

**DOI:** 10.1038/s41598-023-39761-8

**Published:** 2023-08-01

**Authors:** Jianxiao Guo, Liang Zhai, Huiyong Sang, Siyuan Cheng, Hongwei Li

**Affiliations:** 1 Key Laboratory of Surveying and Mapping Science and Geospatial Information Technology of MNR, Chinese Academy of Surveying & Mapping, Beijing, 100036 China; 2 College of Resource Environment and Tourism, Capital Normal University, Beijing, 100048 China; 3 CPC Central Party School (Chinese Academy of Governance), Beijing, 100089 China

**Keywords:** Ecology, Climate-change ecology, Environmental impact

## Abstract

A systematic understanding of the spatio-temporal changes and driving factors in the Qinghai-Tibet Plateau holds significant scientific reference value for the future of ecological sustainable development. This paper utilizes MODIS normalized difference vegetation index (NDVI) and meteorological data to investigate the spatio-temporal changes and driving factors of vegetation coverage in the Qinghai-Tibet Plateau from 2001 to 2020. Methods employed include the dimidiate pixel model, trend analysis, partial correlation analysis, and residual analysis. The results demonstrate a generally fluctuating upward trend in vegetation coverage across the Tibetan Plateau over the past two decades, with spatial expansion occurring from northwest to southeast. Vegetation coverage exhibits a positive correlation with climate factors. Approximately 60.7% of the area showed a positive correlation between vegetation fractional cover (FVC) and precipitation, with 8.66% of the area demonstrating extremely significant (p < 0.05) and significant (p < 0.01) positive correlation. Human activities, on the whole, have contributed to the enhancement of vegetation cover in the Qinghai-Tibet Plateau. The areas where human activities have positively impacted vegetation cover are primarily situated in north-central Qinghai and north of Ngari, while areas experiencing degradation include certain grassland regions in central-eastern Yushu, Nagqu, and Lhasa.

## Introduction

As an important part of terrestrial ecosystems, vegetation coverage and growth condition significantly influences the ecological environment and the capacity for sustainable development^1,2^. The fractional vegetation cover (FVC), which is defined as the ratio of the vertical projection of ground vegetation to the total area of a specific region, serves as a valuable indicator for assessing vegetation dynamics, ecological changes, and surface vegetation growth^3–5^. Hydrothermal conditions are the primary nonbiological factors that determine vegetation characteristics^6^. The underlying connection between FVC and climate factors indicates that FVC may indirectly reflect climate change. Hence, the spatiotemporal variation in FVC proves to be an effective means of elucidating vegetation growth dynamics, monitoring drought stress, and evaluating ecosystem quality in a warming world^7^.

The Qinghai-Tibet Plateau (QTP), situated in Asia, is the largest and highest plateau in the world, often referred to as the "roof of the world" and the "third pole". With a total area exceeding 2.5 million km^2^^8,9^, it serves as an inland plateau in China^8,9^. QTP is a sensitive region in terms of climate change and an ecologically fragile region, and its ecological change has an important impact on Asia and even the global climate^10^. The distinctive climate and geographical characteristics of the QTP have facilitated the development of diverse ecosystem types, such as forests, shrubs, alpine grasslands, alpine meadows, and alpine deserts. The acquisition of vegetation coverage data and the exploration of its spatial and temporal variations are crucial for assessing ecosystem quality on the QTP.

Previous studies have consistently reported an upward trend in vegetation coverage on the QTP. Li et al*.* observed an increasing trend in QTP's vegetation coverage from 2001 to 2010^11^. Duan et al*.* analyzed NDVI data from 2000 to 2018 and found that alpine meadows, alpine grasslands, and overall vegetation on the QTP exhibited an increasing trend in NDVI during the growing season^12^. Similarly, Zhu et al*.* conducted a comprehensive analysis of time series NDVI data from 2000 to 2018 and confirmed a positive trend in vegetation greenness across the QTP^13^. Additionally, these studies have demonstrated the influence of climate change and anthropogenic factors on vegetation growth. Zhong et al*.* reported an overall increase in vegetation density on the QTP from 1999 to 2014, attributed to short-term warming and increased precipitation^14^. Zhu et al*.* indicated that the positive effect of climate change on QTP’s vegetation is weakening while the negative impact is increasing, with human activities playing an increasingly negative role^15^. Han et al*.* identified a significant correlation between average temperature, total precipitation, and vegetation coverage during the growing season on the QTP, with precipitation being the main controlling factor for vegetation growth^16^. Huang et al*.* found that the dominant influence of climate and human activity varied spatiotemporally, with human-dominated regions being smaller than climate-dominated areas^17^. Sun et al*.* used the geodetector model to analyze the effects of data on the intensity of human activity and weather on the spatial distribution of vegetation NDVI in the QTP region^18^.

In conclusion, vegetation changes are driven by climate change and human activities. Climate change manifests primarily through significant alterations in temperature and precipitation, impacting plant photosynthesis, respiration, and overall growth^19^. Human activities exert both negative and positive influences on vegetation, encompassing urbanization, deforestation, overgrazing, agricultural practices, afforestation, and ecological initiatives^20^.

Thus, by utilizing climate data and long-term series of MODIS NDVI datasets, among other sources, along with the residual trend analysis method, this study has the following objectives: (1) to analyze the spatiotemporal dynamics of vegetation variation on the QTP over the past two decades; (2) to elucidate the primary factors contributing to vegetation changes since 2001; (3) to quantify the influence of climate change and anthropogenic activities on vegetation variation in the QTP. This study enhances our comprehension of the underlying mechanisms driving vegetation changes in plateau regions, while offering valuable insights for vegetation restoration efforts on the QTP.

## Result

### Temporal variation characteristics of FVC and hydrothermal factors

**Figure 1:**
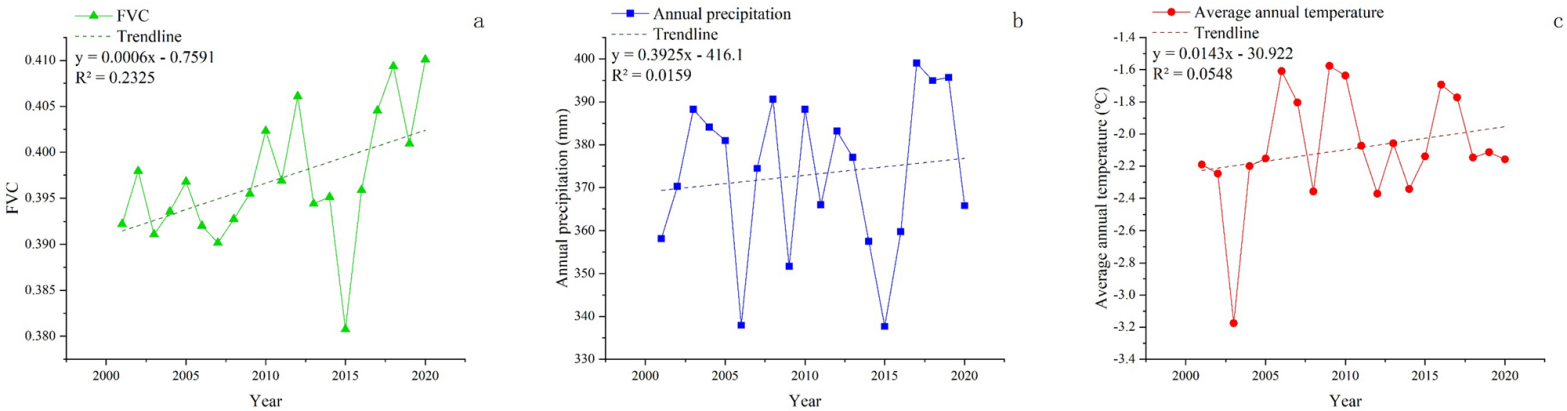
Annual average FVC (**a**), annual precipitation (**b**) and annual average temperature (**c**) change of the QTP from 2001 to 2020.

The average FVC, precipitation, and temperature of the Tibetan Plateau exhibited a general fluctuating upward trend from 2001 to 2020, as shown in Fig. 1a–c.

The annual average FVC reached its maximum value of 0.410 in 2020, while the minimum value was 0.381 in 2015. Precipitation and temperature experienced lower values around 2015 and higher values around 2019. A clear relationship can be observed between vegetation growth and hydrothermal factors, with vegetation coverage varying under different hydrothermal conditions. Overall, the average annual vegetation coverage of the QTP has exhibited a gradual increasing trend over the past 20 years; however, the growth rate has been relatively slow at 0.0006 per year.

### Spatial distribution characteristics of FVC

**Figure 2:**
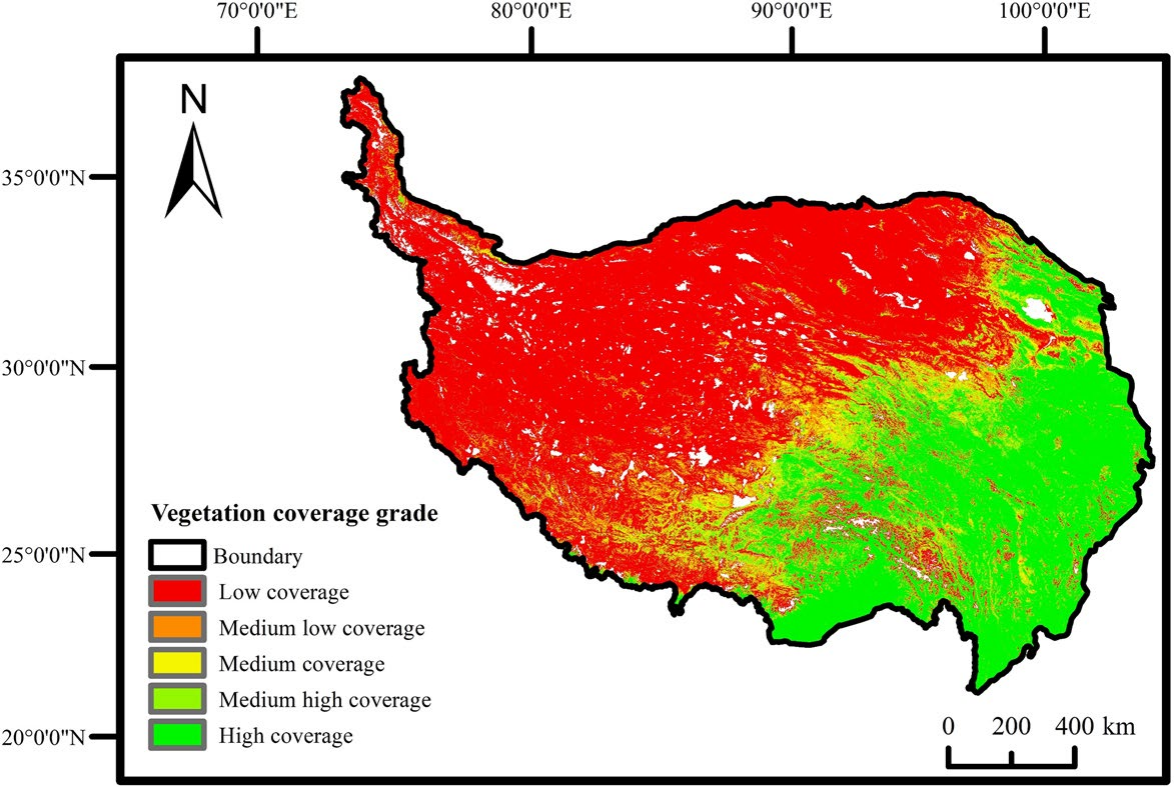
Annual FVC grade distribution of QTP from 2001 to 2020.

To provide a more intuitive representation of vegetation coverage, the FVC was categorized into five levels^14^ (Table 1). The vegetation coverage in the QTP exhibited a distinct spatial pattern of increasing from northwest to southeast in a step-like manner (Fig. 2).

**Table 1:** FVC classification of the QTP.

Grading standard	Grade
0–0.30	Low coverage
0.30–0.45	Medium low coverage
0.45–0.60	Medium coverage
0.60–0.75	Medium high coverage
0.75–1.00	High coverage

Regions with high vegetation coverage were predominantly located in the southeastern areas, characterized by favorable water and heat conditions, encompassing Sichuan Province, Yunnan Province, the southeastern part of Tibet Autonomous Region, the eastern and southern parts of Qinghai Province, and the southern part of Gansu Province. These regions were predominantly covered by coniferous forests and mixed coniferous and broad-leaved forests. Conversely, the regions with low vegetation coverage were situated in the northwestern part of the plateau, characterized by unfavorable water and heat conditions, encompassing the western part of Tibet Autonomous Region, Xinjiang Uygur Autonomous Region, the northwestern part of Qinghai Province, and the western part of Gansu Province. These regions were predominantly covered by grasslands and deserts. This observation indicated a significant correlation between the spatial distribution pattern of vegetation coverage and the hydrothermal conditions.

The statistical analysis revealed that the high and the low vegetation covered area constituted 25.02% and 52.85%,of the total research area, respectively, while the medium and high, the medium, and the medium and low covered area accounted for 7.30%, 6.30%, and 8.53%,respectively.

In order to investigate the spatial evolution characteristics of vegetation coverage on the QTP in more detail, we employed the Theil-Sen Median trend analysis method and the Mann–Kendall significance test method to analyze the trend of variation in vegetation coverage.

**Figure 3:**
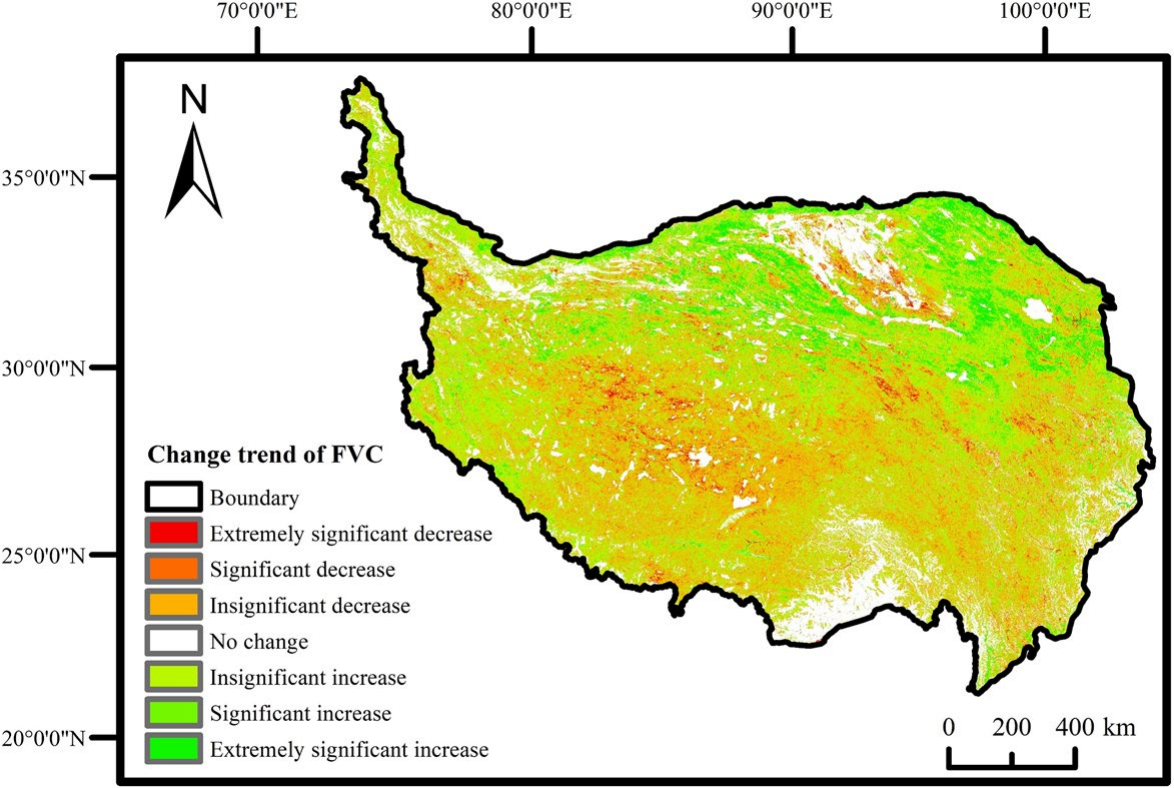
Change trend of FVC on QTP from 2001 to 2020.

The results, as depicted in Fig. 3, revealed that the area with a highly significant increase and significant increase in vegetation coverage constituted 11.94%. This area was predominantly located in the northern region, encompassing the Xinjiang Uygur Autonomous Region (including the Kunlun Mountain system and Aljinshan Mountain system), Gansu Province (Qilian Mountain system), and the northern part of Qinghai Province (Bayankra Mountain system and the vicinity of Qinghai Lake). Conversely, the area with a highly significant decrease and significant decrease accounted for 3.94% of the total research area and was primarily concentrated in the central Tibet Autonomous Region and southwestern Qinghai Province.

### Partial correlation between FVC and precipitation

**Figure 4:**
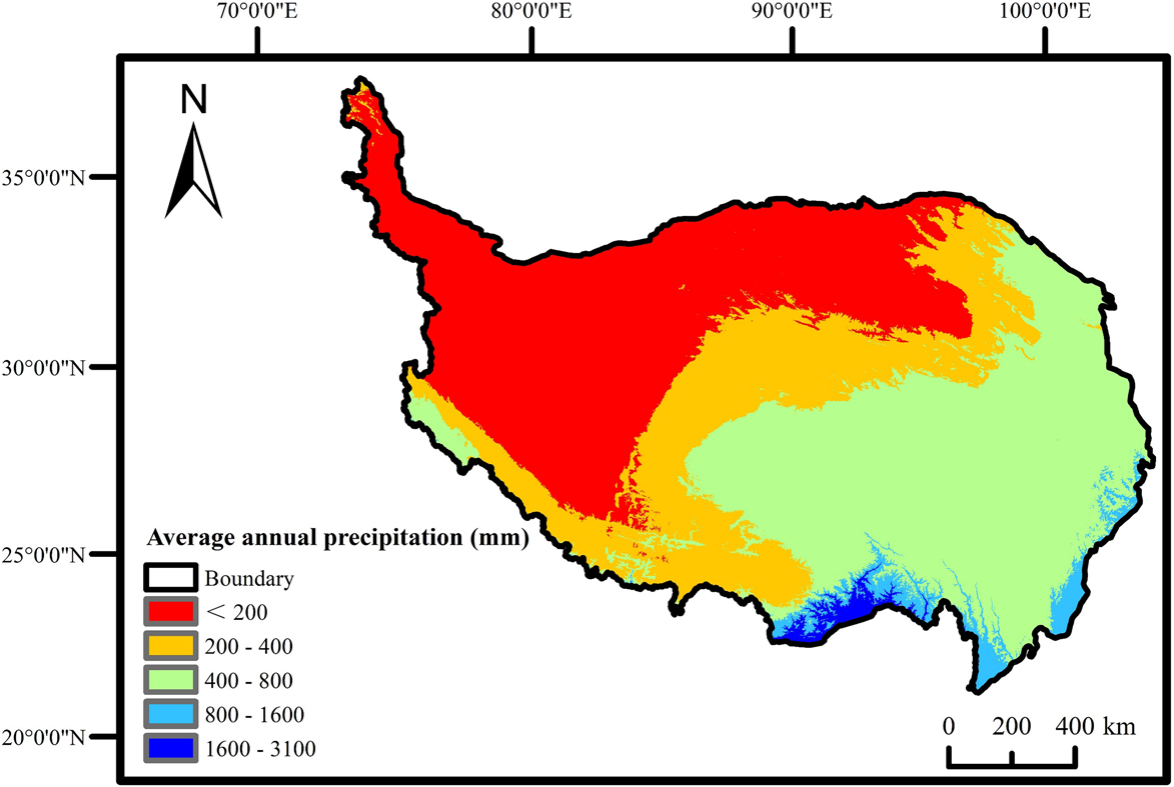
Average annual precipitation change on the QTP from 2001 to 2020.

The spatial distribution of precipitation in the QTP exhibited a gradual increase from northwest to southeast, aligning closely with the distribution pattern of vegetation coverage (Fig. 4).

**Figure 5:**
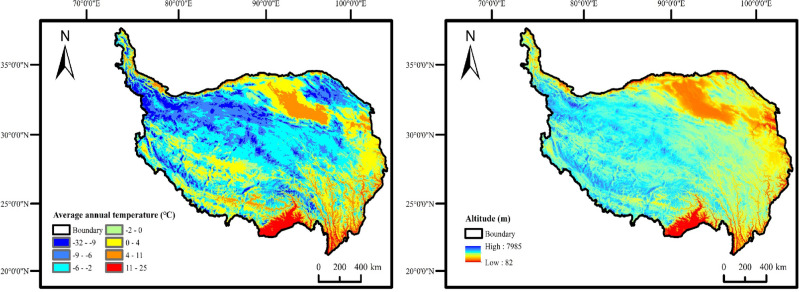
Partial correlation coefficient and significance between FVC and precipitation on the QTP from 2001 to 2020.

Following the partial correlation analysis between vegetation coverage and precipitation, we derived a spatial distribution map illustrating the partial correlation coefficient and the significance of the correlation between FVC and precipitation on the QTP from 2001 to 2020 (Fig. 5).

According to the statistical analysis, 60.7% of the research area exhibited a positive correlation between FVC and precipitation. Among these, 8.66% of the area displayed a highly significant and significant positive correlation, encompassing the grassland areas in Gansu Province, the grassland and meadow areas in southern Qinghai Province, as well as the alpine vegetation, grassland, and meadow areas in central Tibet Autonomous Region. The remaining 39.3% of the area exhibited a negative correlation. Of this portion, a small proportion (1.7%) exhibited a highly significant and significant negative correlation, while 33.5% exhibited an insignificant negative correlation. This included the southeast of Qinghai Province, the Chai Damu Basin, Yunnan Province, and the southeast of Tibet Autonomous Region.

### Partial correlation between FVC and temperature

**Figure 6:**
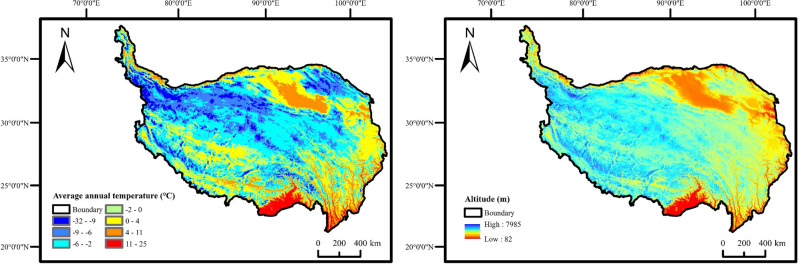
Distribution map of annual average temperature and DEM on Qinghai-Tibet Plateau.

The temperature in the QTP was strongly influenced by altitude (Fig. 6), with temperatures decreasing as altitude increases.

**Figure 7:**
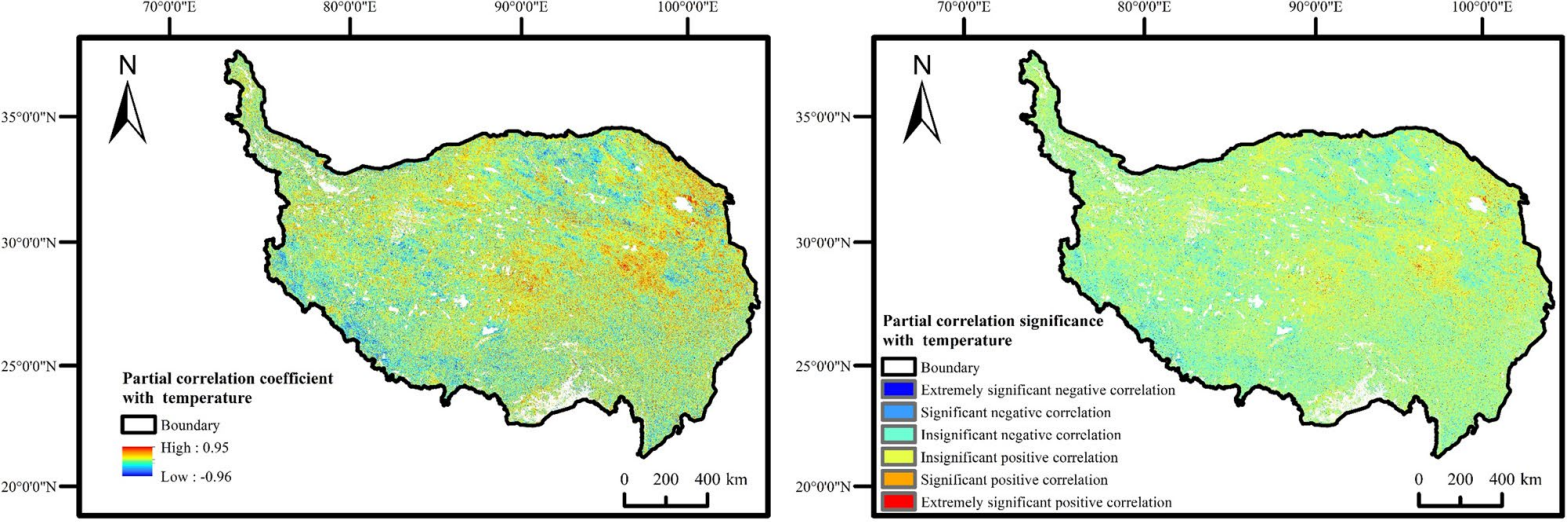
Partial correlation coefficient and significance between FVC and temperature on the QTP from 2001 to 2020.

By analyzing the spatial distribution of the partial correlation coefficient and significance between vegetation coverage and temperature from 2001 to 2020 (Fig. 7), it revealed that 52.8% of the area exhibited a positive correlation between vegetation coverage and temperature. This positive correlation was primarily observed in most parts of Qinghai Province and the northwest of Tibet Autonomous Region (adjacent to Xinjiang). Among these regions, 2.22% exhibited an extremely significant and significant positive correlation, while 50.58% exhibited an insignificant positive correlation. Additionally, 47.2% of the area displayed a negative correlation, predominantly in the southwest of Tibet Autonomous Region. Of this portion, 2.86% exhibited an extremely significant and significant negative correlation, while 44.34% exhibited an insignificant negative correlation.

### Impact of human activities on FVC

**Figure 8:**
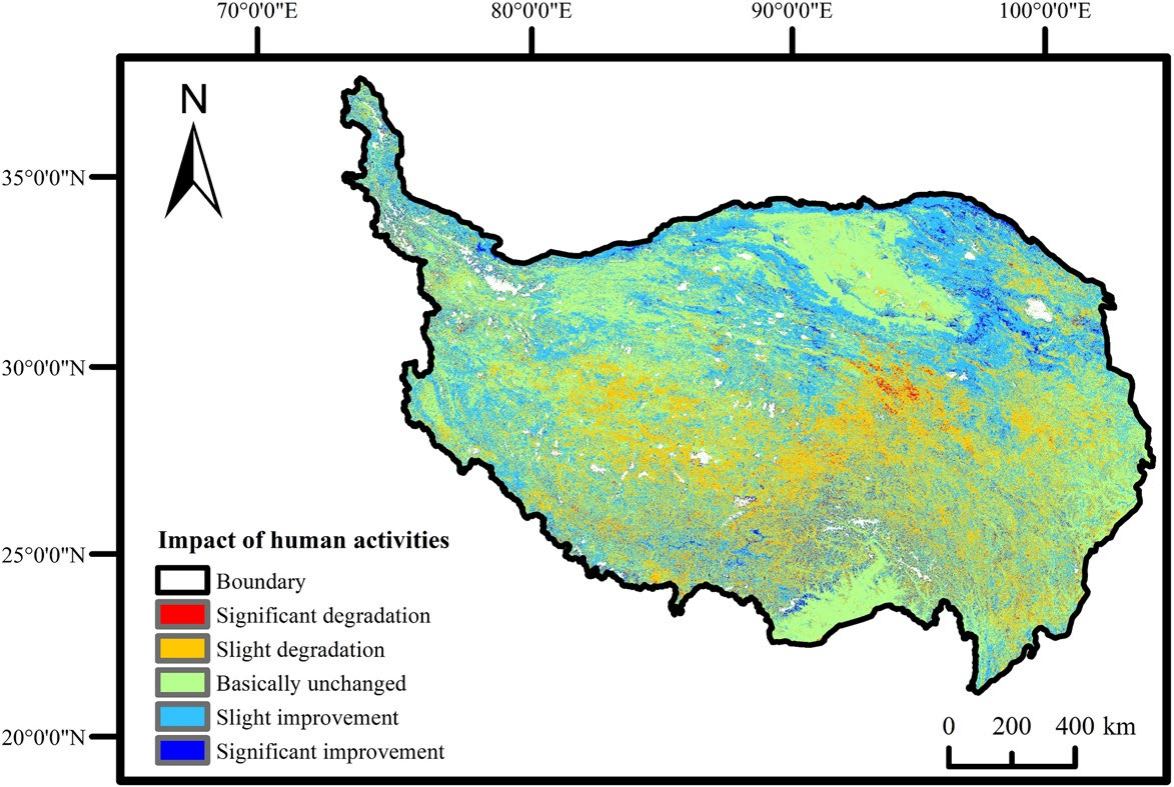
FVC change trend affected by human activities.

Residual analysis was employed to isolate the influence of human activities on FVC (Fig. 8).

**Figure 9:**
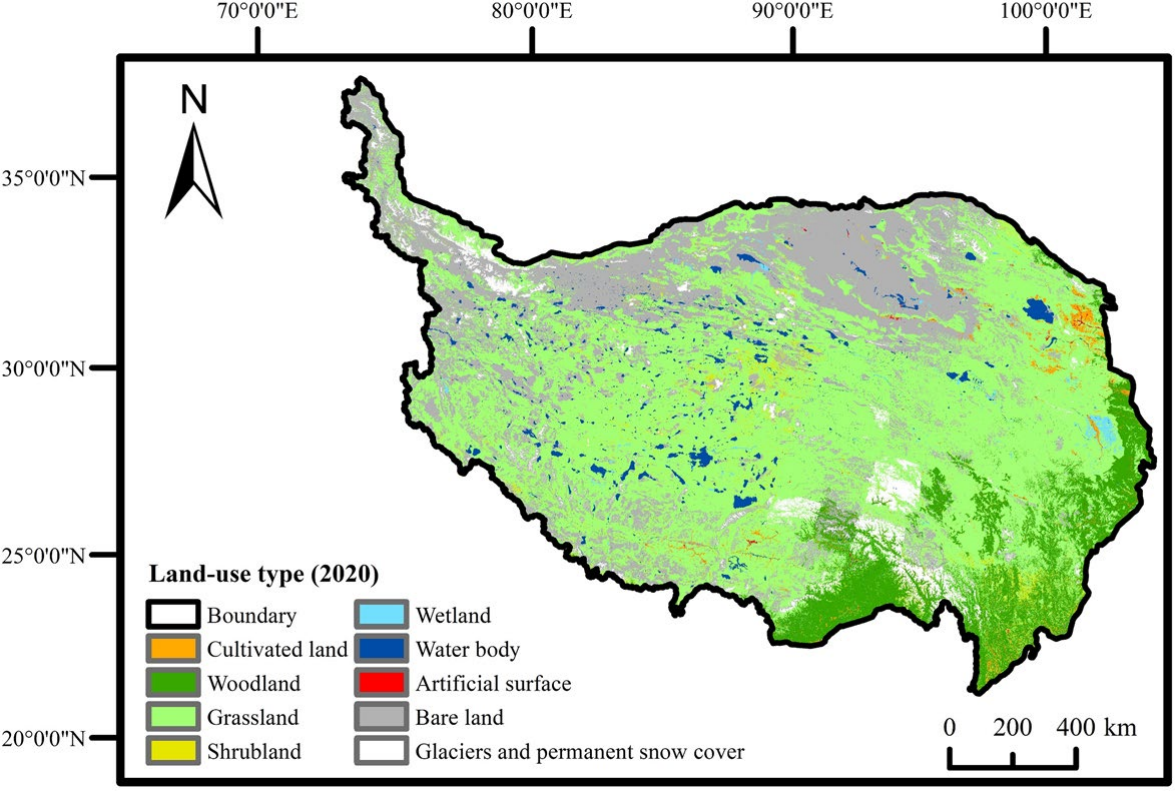
Map of land use types on the QTP.

The statistical analysis revealed that 39.19% of the area experienced improvement due to human activities, while 34.12% remained relatively unchanged, and 26.69% experienced degradation. Consequently, over the past 20 years, human activities have enhanced the overall vegetation coverage of the QTP, and fostered vegetation growth. With respect to land use type (Fig. 9), the areas that experienced improvement were primarily located in the grasslands of Xinjiang, the central and northern areas of Qinghai, cultivated areas in Xining, the northern and southwestern areas of Ali, the southwestern areas of Shigatse, and Nujiang. Areas with minimal change predominantly consisted of bare lands and forest lands, such as the Qaidam Basin in the Hercynian region, and forest lands in the southern parts of Shannan and Nyingchi. Regions exhibiting a detrimental impact were mainly concentrated in grasslands of Nagqu and Yushu, the eastern part of Guoluo, and parts of Qamdo, Diqing, Liangshan, etc. It indicated a relatively severe degradation trend in human-altered surfaces.

## Discussion and conclusion

### Discussion

**Figure 10:**
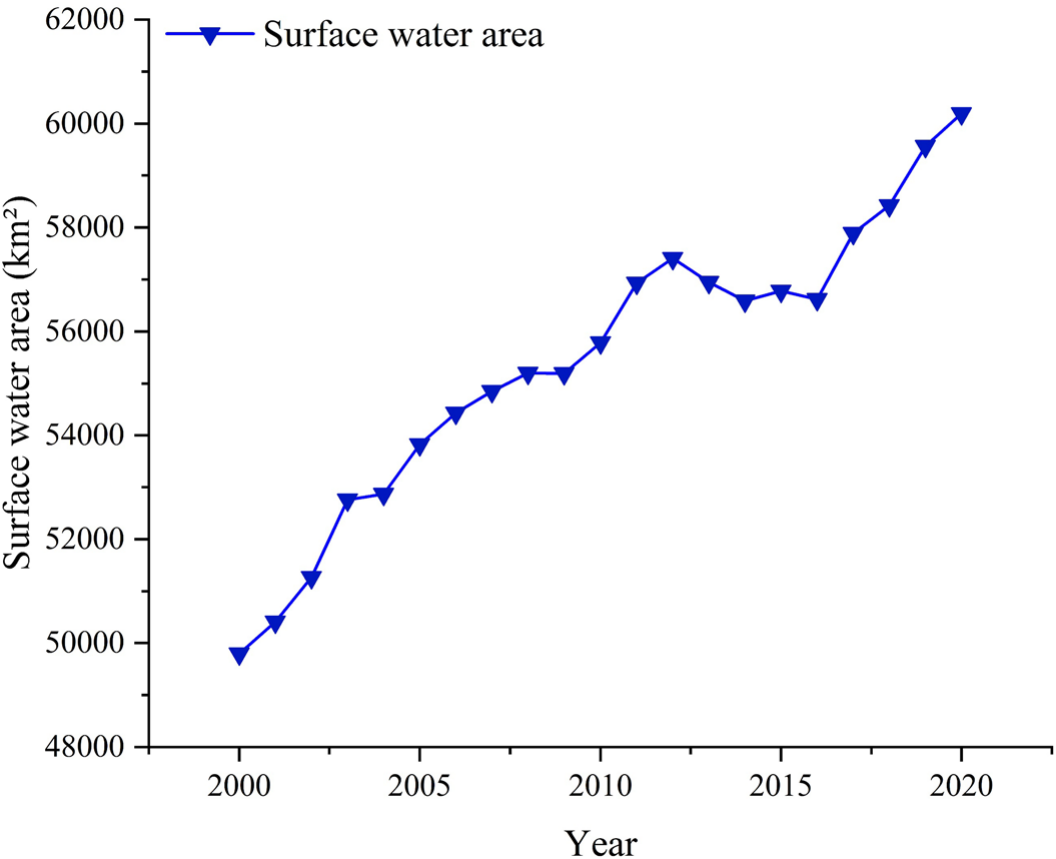
Interannual variation of total surface water area in Tibet and Qinghai from 2001 to 2020.

The vegetation coverage in the QTP exhibited a consistent overall improvement, with a spatial distribution that increased from the northwest to the southeast. This distribution pattern aligns with the prevailing climatic conditions in the QTP. The northwestern region experienced less favorable climatic conditions for vegetation growth compared to the southeastern region. The trend analysis revealed that the vegetation coverage in the QTP has generally improved, but local areas also experienced degradation. These findings aligned with a previous study^21–23^. Additionally, using the Google Earth Engine (GEE) platform, we acquired the total surface water areas of Tibet and Qinghai from 2001 to 2020 (Fig. 10) through processing the v1.3 dataset of JRC Annual Water Classification History.

It was observed that the areas experiencing a significant or substantial decline corresponded closely to the lake-concentrated regions. In addition, by comparing the vegetation type maps, it showed that the dominant vegetation type in these areas was mainly grasslands. Over time, the expansion of the lake's size led to the inundation of vegetation in that particular area, resulting in a decrease in vegetation coverage. These findings align with previous studies^24,25^. A stable area existed in the southernmost part, characterized by subtropical and tropical mountain coniferous forest vegetation that maintains its dense coverage throughout the year. Notably, the regions exhibiting an improving trend in QTP's vegetation were primarily concentrated in the western and northern parts, while the eastern and southern regions displayed a degrading trend. This discrepancy may be attributed to higher population density and relatively greater human activity intensity compared to the western and northern regions.

Through partial correlation analysis, a significant positive correlation was observed between vegetation coverage of QTP and both precipitation and air temperature across the majority of the study area. Precipitation and temperature exhibited complementary driving effects, with precipitation having a greater impact on vegetation coverage compared to temperature. The northern regions of QTP predominantly consisted of deserts, where vegetation coverage exhibited a positive correlation with temperature, but a negative correlation with precipitation. The data demonstrated the sensitivity of vegetation coverage to the changes in water and thermal conditions. Our findings revealed that the changes in precipitation, particularly in the northern regions, were the primary driver of vegetation degradation, aligning with previous research^26^. The occurrence of low temperature and precipitation levels around 2015 provided a plausible explanation for the observed minimum vegetation coverage during that year. Additionally, the relevant research has noted the occurrence of a super El Niño event in 2015 on QTP, which led to a severe drought across almost the entire plateau^27^. This extreme event further contributed to the lowest vegetation coverage recorded on QTP in 2015. The results demonstrated that favorable hydrothermal conditions played a pivotal role in promoting vegetation growth.

Apart from natural climate factors, human activities significantly influence vegetation growth. Human activities have a more localized impact on vegetation compared to the broad-scale effects of climatic factors, particularly in densely populated regions^28^. The distinctive geographical conditions of QTP render the majority of the plateau unsuitable for human habitation, particularly in the western regions, resulting in human activities being predominantly concentrated in the eastern part of the plateau. Based on the statistical findings, the areas exhibiting improved vegetation coverage due to human activities accounted for 39.19%, primarily concentrated in Xinjiang, central and northern Qinghai, cultivated areas of Xining, northern and southwestern Ali, as well as the eastern and southwestern regions of Shigatse, among others. The degraded area constituted 26.69%, primarily distributed in the grassland regions of central and eastern Yushu, areas with man-made surface coverage in central Tibet (Nagqu, Lhasa), as well as the eastern regions of Guoluo and Xining, among others. The above conclusions were consistent with those of previous studies^28,29^. The Northern Steppe region encompasses numerous snow-covered mountains and glaciers, often situated at high altitudes. The vegetation in this area primarily consists of tundra and other alpine plants. Furthermore, the region has a sparse population, limited urban development, and negligible grazing activities, all of which have minimal impact on vegetation coverage. The degraded areas primarily result from intensive animal husbandry, predominantly affecting grassland and meadow vegetation. The expansion of local animal husbandry has accompanied improved living standards. However, the disorderly management of grazing practices and the absence of timely remedial measures have expedited the degradation of grassland vegetation^30^. Moreover, Xining, the leading contributor to Qinghai Province's GDP, exhibits a significant proportion of man-made surface area. The rapid economic growth, dense population, expanding road construction, and infrastructural development in Xining have resulted in notable human activity footprints that impact vegetation coverage. The favorable impact of human activities on vegetation in the northeastern region of the Tibetan Plateau since 2000 can primarily be attributed to the implementation of four significant ecological projects in China: the Natural Forest Resources Protection Project, the Three Northern Protection Forests Project, the Return of Cultivated Land to Forests Project, and the Grazing Ban Project^15,31^.

### Conclusion

This paper investigated the temporal and spatial variation characteristics of FVC influenced by hydrothermal factors and human activities, providing valuable scientific references for the sustainable ecological development of the Qinghai-Tibetan Plateau (QTP). The findings of this study revealed that vegetation growth on the QTP was influenced by various factors, including temperature, precipitation, and human activities. The main conclusions are as follows:

1. Over the past 20 years, the overall vegetation coverage of the QTP exhibited an upward trend, with greening predominantly observed in the northern regions and degradation primarily observed in the southwestern regions.
2. Vegetation response to climatic factors displayed notable spatial variations: vegetation in the northeastern and southwestern regions exhibited negative correlations with temperature and positive correlations with precipitation, while vegetation in the southeast demonstrated negative correlations with precipitation and positive correlations with temperature. Additionally, vegetation in the northwest displayed positive correlations with both climate factors.
3. Over the past 20 years, human activities had an overall positive effect on the vegetation coverage of the QTP, although significant regional variations existed. In the northeastern part of the plateau, human activities exerted a beneficial impact on vegetation. Nevertheless, the adverse effects of human activities on vegetation in the southeastern part of the plateau can not be ignored, and deserve serious attention to prevent further disturbances.

This study focused on two primary climate factors, namely, annual average temperature and annual precipitation. However, given the complexity of geographical systems and human activities, it is necessary to consider other climatic factors. The factors such as relative humidity, solar radiation, and evapotranspiration play significant roles in climate change and are vital for plant development. Climate conditions represent a highly complex system, and the unique geographical location of the Tibetan Plateau renders altitude a crucial factor influencing plant distribution. Subsequent investigations will incorporate altitude, slope, and diverse meteorological conditions to extensively explore the geographic distribution of FVC under the influence of climate change. Moreover, due to the challenges associated with data collection in the QTP, this study solely examined the impact of human activities based on population density, land use, and livestock areas. However, the inclusion of additional data, such as the distribution of tourist attractions and social points of interest, would significantly enhance the accuracy of analyzing the influence of human activities. Consequently, future research can offer more comprehensive multidimensional analysis outcomes, leading to a clearer understanding of the influencing factors that impact vegetation coverage in the region.

## Methods

### NDVI data

The data was obtained from the National Aeronautics and Space Administration (https://ladsweb.modaps.eosdis.nasa.gov/). The dataset comprise MOD13Q1 grid NDVI data products spanning from 2001 to 2020, featuring a spatial resolution of 250 m and a temporal resolution of 16 days. We performed data extraction, splicing, reprojection, cropping, and other preprocessing using the Modis Reprojection Tool and ArcGIS software. The Maximum Value Composite (MVC) method was applied to mitigate the negative influences of cloud, cloud shadow, aerosol, water vapor, visual angle, and solar altitude angle on vegetation coverage calculation^32,33^.

### Climate data

The data was obtained from the National Earth System Science Data Center (http://www.geodata.cn). This dataset^34^ was derived from the global 0.5 climate data provided by CRU (Climatic Research Unit) and the global high-resolution climate data from WorldClim. The dataset was produced in China using the Delta spatial downscaling scheme and validated with 496 independent meteorological observation points. The validation results demonstrated the reliability and accuracy of the climate data. The downscaling process and accuracy evaluation were conducted as follows:

1. The annual mean, maximum, and minimum variables of temperature (TMPs) and precipitation (PRE) for each month were derived from the CRU time series data. The derived climatological dataset has a spatial resolution of 30′, consistent with the CRU dataset.
2. The anomalous time series data for each climate variable were calculated using the CRU time series data and the derived climatological datasets.
  1 $$\begin{array}{c}{TMP}_{an\left(yr,m\right)}={TMP}_{\left(yr,m\right)}-{CRUTMP}_{\left(m\right)}\end{array}$$
  2 $$\begin{array}{c}{PRE}_{an\left(yr,m\right)}=\frac{{PRE}_{\left(yr,m\right)}}{{CRUPRE}_{\left(m\right)}}\end{array}$$
  where ${TMP}_{an\left(yr,m\right)}$ and ${PRE}_{an\left(yr,m\right)}$ are the anomalies for temperatures and precipitation, respectively; ${TMP}_{\left(yr,m\right)}$ and ${PRE}_{\left(yr,m\right)}$ are the absolute temperatures and precipitation values, respectively; ${CRUTMP}_{\left(m\right)}$ and ${CRUPRE}_{\left(m\right)}$ are the 30′ climatology for temperatures and precipitation, respectively; and $m$ and $yr$ correspond to month (January–December) and year, respectively.
3. The 30′ anomaly time series data were spatially interpolated to achieve a higher spatial resolution consistent with the reference dataset from WorldClim.
4. The high-resolution anomaly time series data were converted to absolute climate time series data using WorldClim's reference dataset.
  3 $$\begin{array}{c}{TMP}_{\left(yr,m,res\right)}={TMP}_{an\left(yr,m,res\right)}+{WorldTMP}_{\left(m,res\right)}\end{array}$$
  4 $$\begin{array}{c}{PRE}_{\left(yr,m,res\right)}={PRE}_{an\left(yr,m,res\right)}\times {WorldPRE}_{\left(m,res\right)}\end{array}$$
  where $m$ and $yr$ are defined as above; $res$ represents spatial resolution, i.e., 10′, 5′, 2.5′, and 0.5′; ${TMP}_{\left(yr,m,res\right)}$ and ${PRE}_{\left(yr,m,res\right)}$ are the absolute temperatures and precipitation values with a spatial resolution of res, respectively; ${TMP}_{an\left(yr,m,res\right)}$ and ${PRE}_{an\left(yr,m,res\right)}$ represent anomalies with a spatial resolution of res for temperatures and precipitation, respectively; and ${WorldTMP}_{\left(m,res\right)}$ and ${WorldPRE}_{\left(m,res\right)}$ represent climatology datasets from WorldClim at a spatial resolution of res for temperatures and precipitation, respectively.
5. The original CRU dataset and the downscaled datasets were evaluated using four statistical metrics: Pearson's correlation coefficient (Cor), mean absolute error (MAE), root mean square error (RMSE), and Nash–Sutcliffe efficiency coefficient (NSE). Additionally, the WorldClim data were evaluated at various spatial resolutions by comparing the climatological values of the WorldClim data with observations from the corresponding geographical locations, using the MAE and Cor indices. The sample size corresponds to the number of independent stations.

### Tibetan Plateau boundary data

Downloaded from the National Tibetan Plateau Scientific Data Center^35–39^ (http://data.tpdc.ac.cn).

### Other data

Land use type data is the 2020 version of 30 m global land cover data released by the Ministry of Natural Resources of China.

### Dimidiate pixel model

NDVI exhibits a positive correlation with vegetation coverage. Hence, a pixel binary model wass employed in conjunction with NDVI to estimate vegetation coverage^40,41^. In this paper, we adopted a widely used method to eliminate noise errors. According to the cumulative frequency histogram of NDVI on the whole image, we intercepted the upper and lower threshold values with 5% and 95% confidence levels, and approximated the NDVI values representing bare land and pure vegetation pixels, respectively^42^. The specific formula is as follows:

5 $$\begin{array}{c}FVC=\frac{\left(NDVI-NDV{I}_{\text{soil}}\right)}{\left(NDV{I}_{\text{veg}}+NDV{I}_{\text{soil}}\right)}\end{array}$$

where $FVC$ is the Fractional Vegetation Cover; $NDVI$ is the mixed image element value; $NDV{I}_{\text{soil}}$ is the bare soil image element value; $NDV{I}_{\text{veg}}$ is the pure vegetation image element value.

### Trend analysis

The Theil Sen Medium trend analysis method is a robust nonparametric estimation algorithm that effectively mitigates the influence of outliers in long time series analysis. It represents an improvement over the least squares linear regression method^43,44^. The Mann Kendall test is a nonparametric statistical method introduced by Mann in 1945 and subsequently refined by Kendal and Sneyers^45,46^. One of its advantages is its independence from the assumption of normal distribution for the measured values and linearity of the trend. Moreover, it is unaffected by missing values and outliers^47^.

Sen's slope is estimated by the formula:

6 $$\beta = median\frac{{\left( {FVC_{i} - FVC_{j} } \right)}}{i - j},\;\;\forall i > j$$

where $\beta$ is the slope; $i$ and $j$ are the time series; ${\text{FV}}{\text{C}}_{i}$ and $FV{C}_{j}$ are the FVC values in year $i$ and year $j$, respectively; ${\text{median}}$ is the median function. When $\beta$ >0, FVC has an upward trend; when $\beta$ < 0, FVC has a downward trend.

The results of the Sen trend analysis were tested for significance using the Mann–Kendall method, and the test statistic S was calculated as follows:

7 $$\begin{array}{c}S=\sum \limits_{k=1}^{n-1}\sum \limits_{j=k+1}^{n}S{\text{gn}}\left(FV{C}_{j}-FV{C}_{k}\right);\end{array}$$

8 $$\begin{array}{c}S{\text{g}}{\text{n}}\left({\text{FV}}{\text{C}}_{j}-FV{C}_{k}\right)=\left\{\begin{array}{ll}+1,& \quad \left({\text{FV}}{\text{C}}_{j}-FV{C}_{k}\right) > 0;\\ 0, & \quad ({\text{F}}{\text{V}}{\text{C}}_{j}-FV{C}_{k})=0;\\ -1, & \quad \left({\text{FV}}{\text{C}}_{j}-FV{C}_{k}\right) < 0.\end{array}\right.\end{array}$$

We used the test statistic Z for the trend test.

9 $$\begin{array}{c}Z=\left\{\begin{array}{ll}\frac{S-1}{\sqrt{V{\text{ar}}\left(s\right)}}, & \quad S > 0;\\ 0, & \quad S=0;\\ \frac{S+1}{\sqrt{V{\text{ar}}\left(s\right)}}, & \quad S < 0.\end{array}\right.\end{array}$$

10 $$\begin{array}{c}V{\text{a}}{\text{r}}\left(S\right)=\frac{n\left(n-1\right)\left(2n+5\right)}{18}\end{array}$$

where $S{\text{gn}}$ is the symbolic function, $k$ and $j$ are the time series, and $n$ is the number of monitoring years.

In this paper, using significance levels of P = 0.05 and 0.01, the trend was considered statistically significant when the absolute value of Z exceeded 1.96 and 2.58, corresponding to the 95% and 99% significance levels, respectively. Table 2 presented the methodology used to assess the significance of the observed trend.

**Table 2:** Judgment table of trend significance category.

*β*	*Z*	Trend category
*β* > 0	2.58 < *Z*	Extremely significant increase
	1.96 < *Z* ≤ 2.58	Significant increase
	*Z* ≤ 1.96	Insignificant increase
*β* < 0	2.58 < *Z*	Extremely significant decrease
	1.96 < *Z* ≤ 2.58	Significant decrease
	*Z* ≤ 1.96	Insignificant decrease

### Partial correlation analysis

Partial correlation analysis is a valuable method for examining the relationship between two specific variables while controlling for the influence of a third variable. In partial correlation analysis, the influence of a third variable on the relationship between two variables is removed, allowing for an examination of the correlation between the remaining two variables^48,49^. Using available data on vegetation coverage, precipitation, and temperature, the partial correlation coefficients between vegetation coverage and each of the two climate variables (precipitation and temperature) are computed, and their statistical significance is assessed using t-tests. The specific formula used for calculating the partial correlation coefficients is as follows:

11 $$\begin{array}{c}{r}_{xy,z}=\frac{{r}_{xy}-{r}_{xz}{r}_{yz}}{\sqrt{\left(1-{r}_{xz}^{2}\right)\left(1-{r}_{yz}^{2}\right)}}\end{array}$$

12 $$\begin{array}{c}{r}_{xy,zw}=\frac{{r}_{xy,z}-{r}_{xw,z}{r}_{yw,z}}{\sqrt{\left(1-{r}_{xw,z}^{2}\right)\left(1-{r}_{yw,z}^{2}\right)}}\end{array}$$

13 $$\begin{array}{c}{r}_{xy}=\frac{{\sum }_{i=1}^{n}\left[\left({x}_{i}-\overline{x }\right)\left({y}_{i}-\overline{y }\right)\right]}{\sqrt{{\sum }_{i=1}^{n}({x}_{i}-\overline{x }{)}^{2}{\sum }_{i=1}^{n}({y}_{i}-\overline{y }{)}^{2}}}\end{array}$$

where ${r}_{xy,z}$ represents the partial correlation coefficient between *x* and *y* when the *z* variable is fixed; ${r}_{xy,zw}$ is the second-order partial correlation coefficient between *x* and *y* when the *z* and *w* variables are fixed; ${r}_{xy}$, ${r}_{xz}$, and ${r}_{yz}$ respectively represent the correlation coefficients between *x* and *y*, *x* and *z*, *y* and *z*; *n* is the number of years of monitoring; ${x}_{i}$ and ${y}_{i}$ are respectively the values of the *x* and *y* variables in year *i*, and $\overline{x }$ and $\overline{y }$ are respectively the annual means of *x* and *y*.

Following the computation of each correlation coefficient, a t-test was performed to assess the significance:

14 $$\begin{array}{c}t=\frac{{r}_{xy,zw}}{\sqrt{1-{{r}^{2}}_{xy,zw}}}\sqrt{n-q-2}\end{array}$$

where *n* is the number of monitoring years and *q* is the number of controllable variables.

Using predetermined significance levels of P = 0.05 and 0.01, the t-values exceeding 2.120 and 2.921, respectively, were considered significant at the 95% and 99% confidence levels.

### Residual analysis

This method is an effective approach for distinguishing the effects of climate change and human activities on vegetation dynamics^50^. The impact of human factors is isolated by removing the effects of precipitation and temperature from the long-term time series of vegetation coverage^51^. Considering that the variations in vegetation coverage are primarily driven by climate and human activities, a multiple regression model is developed in this study to estimate vegetation coverage using precipitation and temperature as predictors. The residual represents the discrepancy between the observed and predicted vegetation coverage, capturing the portion attributed to human activities. Lastly, the linear regression method is employed to analyze the temporal trend of the annual residuals. The formula is expressed as:

15 $$\begin{array}{c}FV{C}_{p}=a*pre+b*tem+c\end{array}$$

16 $$\begin{array}{c}\delta =FV{C}_{a}-FV{C}_{p}\end{array}$$

where: $pre$ is annual precipitation data; $tem$ is annual temperature data; a, b, c are regression coefficients; $FV{C}_{p}$ is the predicted value; $FV{C}_{a}$ is the actual Fractional Vegetation Cover calculated from MOD13Q1-NDVI values; $\delta$ is residual. $\delta$ > 0 means human activities play a positive role; $\delta$ < 0 means human activities play a negative role.

The trends of vegetation change under the influence of human activities were classified into five categories: significant degradation^52^ (< − 0.006), slight degradation (− 0.006 to − 0.0006), basically unchanged (− 0.0006 to 0.0006), slight improvement (0.0006 to 0.006), and significant improvement (> 0.006).

## Data Availability

The datasets used and/or analyzed during the current study are available from the corresponding author on reasonable request.
